# Machine Learning Methods of Regression for Plasmonic Nanoantenna Glucose Sensing

**DOI:** 10.3390/s22010007

**Published:** 2021-12-21

**Authors:** Emilio Corcione, Diana Pfezer, Mario Hentschel, Harald Giessen, Cristina Tarín

**Affiliations:** 1 Research Center SCoPE, Institute for System Dynamics, University of Stuttgart, 70563 Stuttgart, Germany; tarin@isys.uni-stuttgart.de; 2 Research Center SCoPE, 4th Physics Institute, University of Stuttgart, 70569 Stuttgart, Germany; diana.pfezer@pi4.uni-stuttgart.de (D.P.); m.hentschel@pi4.uni-stuttgart.de (M.H.); h.giessen@pi4.uni-stuttgart.de (H.G.)

**Keywords:** glucose sensing, surface-enhanced infrared absorption spectroscopy, sensor calibration, machine learning, regression analysis, artificial neural network, Gaussian process regression

## Abstract

The measurement and quantification of glucose concentrations is a field of major interest, whether motivated by potential clinical applications or as a prime example of biosensing in basic research. In recent years, optical sensing methods have emerged as promising glucose measurement techniques in the literature, with surface-enhanced infrared absorption (SEIRA) spectroscopy combining the sensitivity of plasmonic systems and the specificity of standard infrared spectroscopy. The challenge addressed in this paper is to determine the best method to estimate the glucose concentration in aqueous solutions in the presence of fructose from the measured reflectance spectra. This is referred to as the inverse problem of sensing and usually solved via linear regression. Here, instead, several advanced machine learning regression algorithms are proposed and compared, while the sensor data are subject to a pre-processing routine aiming to isolate key patterns from which to extract the relevant information. The most accurate and reliable predictions were finally made by a Gaussian process regression model which improves by more than 60% on previous approaches. Our findings give insight into the applicability of machine learning methods of regression for sensor calibration and explore the limitations of SEIRA glucose sensing.

## 1. Introduction

The reliable detection and identification of specific biomolecules in complex environments have been a long-standing problem in life sciences and promise a variety of potential applications in food process control, environmental monitoring, and health care [1]. The main challenge thereby is that in real world scenarios not only are analyte concentrations very small, but their detection is further disturbed by the extremely large number of additional molecular specimens in the sample. A high sensitivity and selectivity are therefore paramount for any biosensing measurement system. Naturally, this has motivated a number of scientific publications exploring different approaches to the matter. In recent years, the determination of glucose concentrations has been of particular interest. This is—in part—motivated by a potential medical application in the treatment of *diabetes mellitus*, a disease that requires the constant control of a patient’s blood sugar levels [2,3,4]. Given that continuous glucose monitoring devices are approved and in use, research in this area focuses on the development of non-invasive measuring techniques that carry a significantly lower risk of infection [5,6]. On the other hand, however, the detection of glucose can also be considered a case study in fundamental biosensor research. Here, the objective is not necessarily the design of a commercial healthcare product, but the validation and evaluation of an innovative method of measurement through an example application.

Comprehensive reviews regarding the state of the art in the field of glucose sensing have been published by [7,8,9]. Currently, considered approaches include enzyme-based electrochemical biosensors [10], reverse iontophoresis methods [11], and microwave sensing techniques [12], which exploit the correlation between the substrate’s electromagnetic properties and its glucose content. For the latter, the incorporation of artificial so-called “meta-materials” has recently been explored [13,14]. Furthermore, fluorescence-based sensing systems receive much attention due to their extreme sensitivity and their potential for non-invasiveness [15]. In short, they use molecules that absorb and re-emit radiation energy proportional to the glucose concentration in the sample [16]. Another, equally sensitive optical sensor concept exploits the localised surface plasmon, a collective oscillation of the conduction electrons on the surface of a metallic nanostructure which is excited by incoming light. The plasmon’s resonance frequency is dependent not only on the nanostructure’s geometry, but also on the surrounding refractive index, which, in return, is shifted by biomolecular interactions at the surface [17,18]. While this method can be used to detect even single molecules, it is highly complex to distinguish between different analytes. Infrared spectroscopy, on the other hand, notably achieves supreme specificity. Determined by their chemical structure, biomolecules exhibit characteristic vibrational modes, often referred to as spectral fingerprints [19]. Hence, upon electromagnetic irradiation, these specific frequencies are absorbed, which results in a unique spectral absorption pattern. However, the detection of low specimen concentrations proves challenging for standard infrared spectroscopy [20].

### 1.1. SEIRA Glucose Sensing

Surface-enhanced infrared absorption (SEIRA) spectroscopy combines the sensitivity of plasmonic systems and the specificity of infrared spectroscopy [21]. It makes use of the fact that at the plasmon’s resonance frequency electromagnetic near-fields are greatly enhanced. Therefore, if the metallic nanostructure is designed such that its resonance frequency matches the analyte’s molecular fingerprint, their modal coupling amplifies the characteristic vibrational signal by several orders of magnitude, which, in return, enables the detection of lower concentrations [22]. In fact, SEIRA spectroscopy has successfully been applied by Kühner et al. [23] to detect glucose in the presence of fructose in aqueous solutions. Such mixtures are widely used in research as exemplary systems since the presence of monosaccharides with similar physical properties hampers a precise determination of the glucose concentration.

**Figure 1:**
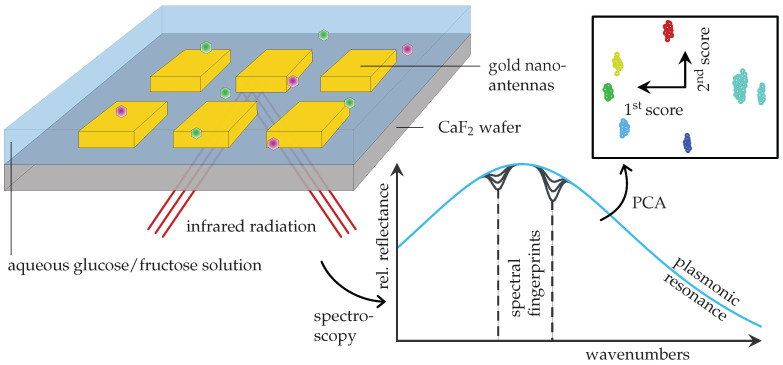
Schematic overview of the SEIRA spectroscopy glucose sensing setup by Kühner et al. [23]: on top, the irradiated reflective flow cell with gold nanoantennas containing an aqueous monosaccharide solution; below, an abstracted plot of the measured spectrum and its principal component decomposition.

The employed SEIRA sensor setup is illustrated in Figure 1.

Incorporated into a reflective flow cell on top of a calcium fluoride (${\mathrm{CaF}}_{2}$) wafer are arrays of linear gold nanoantennas whose geometry was chosen such that their plasmonic resonance coincides with the characteristic molecular modes of glucose and fructose. Therefore, their enhanced vibrational fingerprint is encoded in the measured spectrum of the reflected infrared beam, which is schematically depicted in the lower right. Here, the blue curve represents the pure plasmonic resonance while the dips at the highlighted spectral fingerprints are caused by the presence of monosaccharide molecules [24]. Generally speaking, the larger these dips, the higher the concentration. In order to extract this critical pattern from the data and to quantify it, the authors perform a principle component analysis (PCA) [25], which, as shown in the top right corner, reveals distinct clusters for different concentrations of glucose and fructose in the aqueous solution. This does not only confirm that these two monosaccharides indeed have unique spectral fingerprints despite their similar chemical structure, but that SEIRA is specific enough to distinguish between them.

### 1.2. SEIRA Glucose Sensing—Inverse Problem

**Figure 2:**
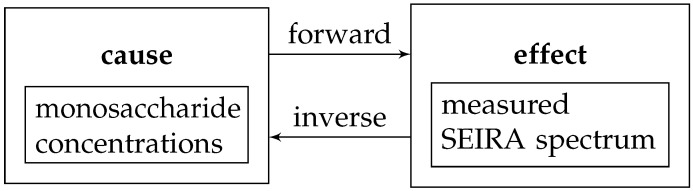
Flow chart visualising the forward/inverse problem of sensing with SEIRA spectroscopy of sugary solutions as an application example.

The present paper directly advances the efforts of the publication cited above. The overall challenge addressed here can be referred to as the inverse problem of sensing (also referred to as sensor calibration), as schematically outlined in Figure 2.

While Kühner et al. [23] studied the so-called forward mapping, that is, the influence different parameter values have on the system’s output, the inverse problem, on the other hand, deals with the prediction of the underlying physical properties based on some given measurements. For the application scenario at hand, this implies the reliable reconstruction of the precise glucose and fructose concentrations in aqueous solutions from the SEIRA sensor data. A first solution for this was proposed by Schuler et al. [26]. Instead of a PCA, the authors perform an asymmetric least squares smoothing (ALSS) baseline correction [27] to remove the plasmonic background and isolate the pure vibrational spectrum with peaks at the characteristic wavenumbers. Their heights directly correlate with their associated monosaccharide concentrations. Subsequently, a superposition of quadratic basis functions is optimised over both the glucose and fructose concentration to match the fingerprint amplitudes. Eventually, negative results are set to zero and the average from 30 samples is drawn to compute the final prediction. Note that this method has several drawbacks: First, and foremost, taking only the relative reflectance at the precise fingerprint wavenumbers into consideration neglects spectrum-shifting disturbances and thus compromises the robustness of the prediction. On top of that, the ALSS-algorithm returns a different baseline to be eliminated from the spectrum for each measurement which clearly restricts the comparability. Additionally, removing a fitted Fano or Lorentzian shaped baseline instead is also not an expedient solution since such a theoretic model deviates heavily from the actual measurements. Finally, the authors assume the contribution of pure water to be constant, which, however, is not the case. This is further elaborated on in Section 3.1. Overall, Schuler et al. [26] propose an optimisation-based solution to the inverse problem. However, since a functional dependence is to be determined or at least estimated, this problem can also be approached via a regression analysis. Regression analysis is a statistical process where an unknown function mapping an independent input vector $x\in R^{p}$ onto the dependent output vector $y\left(x\right)\in R^{q}$ is approximated by fitting a pre-defined model

(1) $$\begin{array}{cc} f:\; & R^{p}\times R^{r}\to R^{q} \end{array}$$

as closely as possible to a given set of *N* observations

(2) $$\begin{array}{cc} D & =\left\{\left(x_{i},\;y_{i}\right)|\;i=1\ldots N\right\} \end{array}$$

by minimising the norm of the model prediction error e=y−f(x,β) over the parameter vector $\beta \in R^{r}$ such that

(3) $$\begin{array}{c} y=f(x,\;\beta )+e. \end{array}$$

Once the model is optimised, it can be employed to estimate the system output for any given input vector. Thereby, the determining factor for the quality of the prediction is the underlying function *f* which can either be chosen based on a physical system model or as a universal ansatz function. While the first approach reduces the regression to a parameter identification, the latter belongs to the field of machine learning as it is purely data-driven and treats the system as a black box.

### 1.3. Contribution

At the time of writing, to the authors’ best knowledge, there is no adequately accurate mathematical replica of the SEIRA sensing process or sensor available. In addition, the obtained measurement data exhibit a high variance, which overall suggests that model-based approaches are unsuited to solve the inverse problem of SEIRA spectroscopy. Therefore, within the scope of this paper, we propose several advanced supervised machine learning algorithms for regression analysis to reliably predict the output vector

(4) $$\begin{array}{c} y=\left[\begin{array}{c} c_{\mathrm{gluc}}\\ c_{\mathrm{fruc}} \end{array}\right]\in R^{q=2} \end{array}$$

containing the concentrations of both glucose $c_{\mathrm{gluc}}$ and fructose $c_{\mathrm{fruc}}$ in aqueous solutions.

The contribution of this paper is twofold: Most importantly, we analyse and compare the performance of the different machine learning methods in order to evaluate their applicability in the field of sensor calibration. As this is fundamental research, we expect our findings here to be generalisable and potentially transferable to other biosensing systems. Moreover, we determine the limitations of the surface-enhanced infrared absorption spectroscopy measurement setup by Kühner et al. [23] from a signal processing perspective to support the further development of the sensor.

This paper is structured as follows: First, we introduce the utilised regression algorithms, covering both their theoretical backgrounds as well as their known applications and advantages. Next, we delineate the experimental setup and present the measurement data, as well as our pre-processing routine in detail. Finally, the different regression strategies are implemented and their performance is showcased for solutions of varying monosaccharide concentrations. This allows for a thorough evaluation and discussion.

## 2. Regression Methods in Machine Learning

In total, two different regression strategies are employed and compared. In this section, both are presented in detail and their application is motivated.

### 2.1. Cascade-Forward Neural Network

Artificial neural networks (ANN) are powerful universal function approximators that are loosely designed after the synapses in the human brain. As such, they are formed by interconnected basic units of computation, referred to as neurons, which are usually structured in layers. The first one, the input layer, is passed the independent data vector $x\in R^{p}$, whereas the last one, the output layer, returns the estimate for the dependent variable $y\left(x\right)\in R^{q}$. Between them, there can be arbitrarily many so-called hidden layers. In a fully connected feed-forward network, all neurons in one layer are connected to those in the following one. These connections define input/output relations between the neurons and are assigned a weight: A neuron’s scalar input is defined as the weighted sum over its connected neurons’ outputs and a bias term. Accordingly, the input vector of the kth layer is given by

(5) $$\begin{array}{c} W^{\left(k\right)}z^{\left(k-1\right)}+b^{\left(k\right)}, \end{array}$$

where $z^{\left(k-1\right)}$ denotes the output vector of the (k−1)th layer, $W^{\left(k\right)}$ the matrix of weights and $b^{\left(k\right)}$ the vector of biases. The layer’s output vector, in return, is computed by applying a nonlinear activation function ϕ:R→R element-wise to its input vector (5). Due to this incorporated nonlinearity, a neural network with sufficiently many hidden neurons can be fit arbitrarily accurately to any function (universal approximation theorem) by numerically optimising the network’s weights and biases with respect to a given set of labelled measurement data D. This process is referred to as training and usually accomplished via some gradient-based algorithm, which is to avoid local minima and an overfitting of the network. In short, artificial neural networks essentially are particularly structured ansatz functions for regression analysis with an extremely high number of optimisation parameters [28].

**Figure 3:**
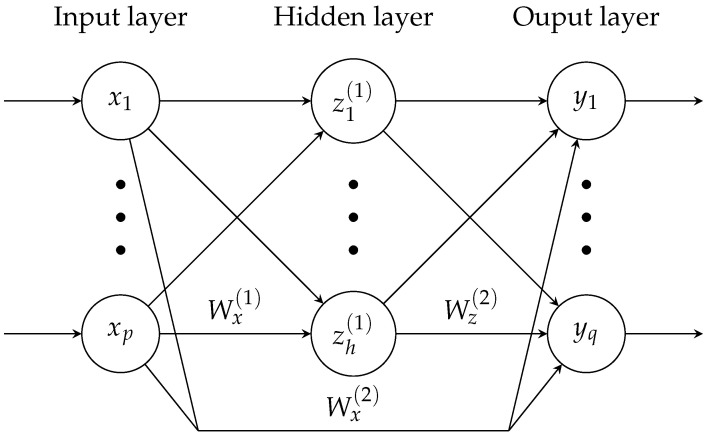
Graphical representation of a fully connected cascade-forward neural network with one hidden layer. The network features *p* input nodes $x_{1\ldots p}$, *h* hidden neurons $z_{1\ldots h}^{\left(1\right)}$ and *q* outputs $y_{1\ldots q}$. The respective weight matrices are denoted by $W_{.}^{\left(\;\cdot \;\right)}$, while the bias terms are not explicitly shown.

A special class of ANNs are cascade-forward neural networks (CFNN), which feature additional connections between the different layers. More precisely, each layer, including the input layer, is connected not only to its immediate successor, but to all successive layers in the network. This way, linear and nonlinear relationships can be modelled separately and the improved data distribution increases the network’s ability to generalise [29]. The standard topology of a cascaded network with one hidden layer containing *h* neurons is schematically depicted in Figure 3.

In this case, compared to a simple feed-forward network, the CFNN only differs in its direct paths from the inputs *x* to the outputs *y*, weighted by the matrix $W_{x}^{\left(2\right)}\in R^{q\times p}$. The matrices $W_{x}^{\left(1\right)}\in R^{h\times p}$ and $W_{z}^{\left(2\right)}\in R^{q\times h}$, on the other hand, weight the connections between the input and the hidden layer and the hidden and the output layer, respectively. Overall, this network architecture yields the output equation

(6) $$\begin{array}{cc} f & =W_{x}^{\left(2\right)}x+W_{z}^{\left(2\right)}z^{\left(1\right)}+b^{\left(2\right)}, \end{array}$$

where

(7) $$\begin{array}{cc} z^{\left(1\right)} & =\phi \left(W_{x}^{\left(1\right)}x+b^{\left(1\right)}\right),\;z^{\left(1\right)}\in R^{h} \end{array}$$

describes the activation levels of the neurons in the single hidden layer and $b^{\left(1\right)}\in R^{h},\;b^{\left(2\right)}\in R^{q}$ denote the biases. As training the network implies optimising over its weights and biases, combining these to the parameter set

(8) $$\begin{array}{cc} \beta & =\left\{W_{x}^{\left(1\right)},\;W_{x}^{\left(2\right)},\;W_{z}^{\left(2\right)},\;b^{\left(1\right)},\;b^{\left(2\right)}\right\} \end{array}$$

recovers the structure of the original regression model (3).

As a staple of modern machine learning, artificial neural networks have, amongst other things, been used successfully to solve the inverse problem of sensing. For instance, Ref. [30] advocate the use of ANNs to linearise nonlinear sensor outputs and point out in particular their convenient hardware implementation. Ref. [31], in return, present a neural network as calibration method for a force/torque sensor and achieve good results. Calibration strategies based on neural networks have even been proposed for pressure [32] and temperature [33] sensors to overcome hysteresis and the lack of linearity in time with remarkable performances. The application of neural networks in the field of biosensing has been discussed in the literature as well: Ref. [34] successfully employed an ANN as multivariate calibration model for an amperometric biosensor, while Ref. [35] reported highly promising results by combining surface-enhanced Raman spectroscopy biosensors with neural network algorithms. To estimate the glucose levels in human blood by processing the measurement signal of a non-invasive near-infrared spectroscopy (NIRS) sensing system, Ref. [36] proposes an inverse function delayed neural network, while Ref. [37] employs a nonlinear stacked auto-encoder deep neural network to the same end. However, it is most common in this context is to train neural networks as models of the forward mapping, i.e., to predict the resonance spectrum either from the molecular structure [38] or from the plasmonic geometric parameters [39].

### 2.2. Gaussian Process Regression

Gaussian process modelling is a non-parametric Bayesian approach towards regression and classification problems. Its core idea is, that instead of specifying a parametric model function f(x,β), it is assumed that *f* is distributed as a Gaussian process

(9) $$\begin{array}{cc} f(x) & ∼\mathrm{GP}\left(\mu \left(x\right),\;k\left(x,\;x^{\prime }\right)\right). \end{array}$$

A Gaussian process GP defines a probability distribution in the function space and is completely determined by a mean

(10) $$\begin{array}{cc} \mu (x) & =E\left[f(x)\right] \end{array}$$

and a covariance or kernel function

(11) $$\begin{array}{cc} k(x,\;x^{\prime }) & =E\left[\left(f(x)-\mu (x)\right)\left(f\left(x^{\prime }\right)-\mu \left(x^{\prime }\right)\right)\right]. \end{array}$$

**Figure 4:**
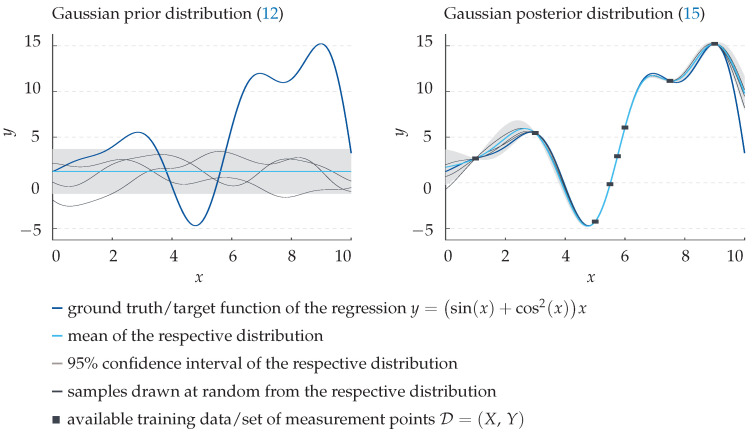
Visualisation of the working principle of a Gaussian process regression with an example target function. Note that both the input x∈R, as well as the dependent output y∈R in this example are dimensionless. (**Left panel**) Gaussian prior distribution (12). (**Right panel**) Gaussian conditional posterior distribution (15) approximating the underlying target function *y* from given measurement points D=(X,Y).

The mean μ(x) simply corresponds to the distribution’s expected value and can therefore be regarded as a naive guess for the underlying function *f*. The kernel $k(x,\;x^{\prime })$, on the other hand, describes the covariance or similarity between the function values of two points *x* and $x^{\prime }$ and thus encodes prior assumptions about the properties of *f*, such as its smoothness and periodicity. Evaluating the Gaussian process for some arbitrary input vector $x^{*}$ implies sampling from the multivariate normal distribution

(12) $$\begin{array}{c} f\left(x^{*}\right)∼N\left(\mu \left(x^{*}\right),\;k\left(x^{*},\;x^{*}\right)\right) \end{array}$$

commonly referred to as prior. This is showcased in the left panel of Figure 4.

The fine black curves represent three samples drawn at random from this distribution. Note that their shape is determined exclusively by the chosen mean and kernel function. However, given a set D=(X,Y) of known training data with

(13) $$\begin{array}{c} \begin{array}{cc} X & =\left[\begin{array}{ccc} x_{1} & \ldots & x_{N} \end{array}\right]\in R^{p\times N},\\ Y & =\left[\begin{array}{ccc} y_{1} & \ldots & y_{N} \end{array}\right]\in R^{q\times N}, \end{array} \end{array}$$

this information can be incorporated and the Gaussian prior can be expanded to the joint distribution

(14) $$\left[\begin{array}{c} Y\\ f(x^{*}) \end{array}\right]∼N\left(\left[\begin{array}{c} \mu (X)\\ \mu (x^{*}) \end{array}\right],\;\left[\begin{array}{cc} K+\sigma_{n}^{2}I & k_{*}\\ k_{*}^{⊤} & k(x^{*},\;x^{*}) \end{array}\right]\right)$$

where $\sigma_{n}^{2}$ is the variance of some additive zero mean measurement noise and K=K(X,X), $k_{*}=K\left(X,\;x^{*}\right)$ denote the covariance matrices between all training points and all pairs of training and test points, respectively. By conditioning the joint prior on the previous observations, the multivariate posterior

(15) $$\begin{array}{c} \begin{array}{cc} f(x^{*}) & |\;X,Y,x^{*}∼N\left({\bar{f}}^{*},\;\mathrm{cov}\left(f^{*}\right)\right)\\ {\bar{f}}^{*} & =\mu \left(x^{*}\right)+k_{*}^{⊤}{\left[K+\sigma_{n}^{2}I\right]}^{-1}\left(Y-\mu (X)\right)\\ \mathrm{cov}(f^{*}) & =k\left(x^{*},\;x^{*}\right)-k_{*}^{⊤}{\left[K+\sigma_{n}^{2}I\right]}^{-1}k_{*} \end{array} \end{array}$$

is obtained which describes the probability distribution of the function value $f(x^{*})$ given the training data set D. As can be seen in Figure 4 on the right, in contrast to the prior, samples from the posterior approximate the underlying function (dark blue) quite well, even for a small number of measurement points (black squares). Hence, as predictive output of the regression model, one can simply take the posterior mean ${\bar{f}}^{*}$ (light blue), a finite sum of weighted kernel functions centered around the measurement points *X* [40]. Note that, unlike ANNs, a Gaussian process regression does not require any numerical training but instead uses all available data explicitly for each single prediction. While this may cause a significantly higher computation time, training-based effects, like overfitting the regression model, do not occur. Moreover, the covariance $\mathrm{cov}(f^{*})$ inherently provides a benchmark for the quality of the prediction, as shown by the light grey 95% confidence intervals in Figure 4.

The applicability of Gaussian process regression to the inverse problem of sensing has been demonstrated in the literature: In fact, both the estimation of the gas concentration a MOX sensor is exposed to [41], as well as the output quantification of a flexible tactile sensor afflicted by measurement noise and hysteresis are handled equally well [42]. On top of that, Ref. [43] points out the robustness against drift environments achieved by calibrating a chemiresistor sensor via a GPR model. In addition, based on GPR, several calibration models for near-infrared spectroscopic sensing have been developed and employed to predict single chemical properties [44,45,46]. Here, the authors specifically emphasise the improved performance over traditional regression methods. However, regarding glucose sensing, the employment of Gaussian process regression is currently limited to predictions of the blood glucose levels based on physical activity data [47] or general categorical information [48].

**Remark** **1.** *At the time of writing, to the authors’ best knowledge, neither a Gaussian process regression model, nor any kind of artificial neural network has been proposed as a possible solution to the inverse problem of surface-enhanced infrared absorption spectroscopy featuring gold nanoantennas. Besides, the findings of such a comparative study could also prove insightful for the calibration of other biosensing systems.*

## 3. Experimental Setup

As this paper builds upon the work of the aforementioned publications by Kühner et al. [23] and Schuler et al. [26], we employ exactly the same sensor setup as described in Section 1, measuring by SEIRA spectroscopy an analyte’s (dimensionless) relative reflectance $r\left(\tilde{\nu }\right)\in \left[0,\;1\right]$ in the spectral range from ${\tilde{\nu }}_{\min}=900\;{\mathrm{cm}}^{-1}$ to ${\tilde{\nu }}_{\max}=1300\;{\mathrm{cm}}^{-1}$. After each measurement, the sensor flow cell is flushed clean to remove any residual molecules. Technical details and specifications regarding the used equipment are listed in Appendix A.

Overall, measurements are performed on pure water, aqueous solutions containing a single monosaccharide, i.e., either glucose or fructose, and aqueous solutions containing both monosaccharides. The respective concentrations thereby range from 0 g/L to 60 g/L. With 69 samples, most measurements are performed on pure water, which allows for establishing a reliable zero basis for the regression. The exact number of analysed samples with the corresponding monosaccharide concentration compositions are listed in the two tables below: Table 1 provides information on the single monosaccharide measurement cycles, where the experimental setup is identical for glucose and fructose, while Table 2 gives insight into the analysis of the double monosaccharide solutions.

For each sample, 25 measurements are recorded. This data are subsequently split up: 80% of the spectra are used to train the various regression models introduced in Section 2 (training data), while the remaining 20% are used to evaluate the prediction accuracies (test data). This approach is considered state of the art in machine learning.

A recorded reflectance spectrum $r(\tilde{\nu })$ is not directly fed into a regression algorithm as input vector $x\in R^{p}$. Instead, it is first subject to the pre-processing routine presented in the following.

### 3.1. Data Pre-Processing

**Figure 5:**
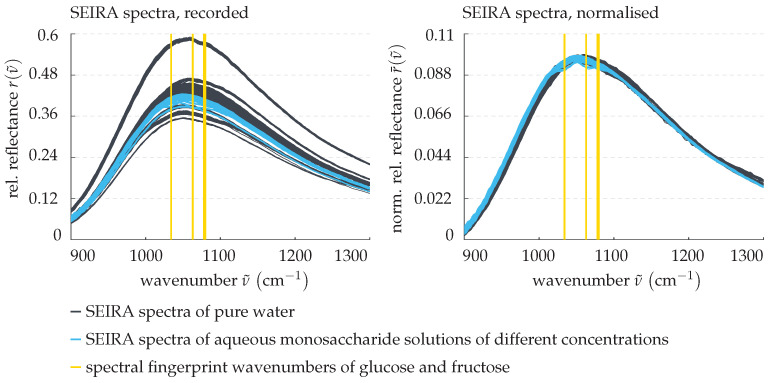
Pre-processing step 1: normalisation of the recorded SEIRA spectroscopy sensor data. (**Left panel**) raw measured spectra. (**Right panel**) spectra normalised with respect to the Euclidean norm.

Figure 5 shows on the left some example measurement data as obtained directly from the SEIRA spectroscopy sensor.

The spectra depicted in black were recorded for measurements performed on pure water, while the blue spectra belong to some aqueous monosaccharide solutions of different concentrations. Note that in this section the figures serve as qualitative visualisation and are not purposed for a quantitative analysis. As can be seen, in both cases, the spectral baselines are affected by a notably varying scaling. This can be attributed to fluctuations in the transmitted energy of the measurement signal caused by external interferences and disturbances. In order to compensate for this, the measured spectra are normalised with respect to the Euclidean norm such that

(16) $$\begin{array}{c} \bar{r}\left(\tilde{\nu }\right)=\frac{r(\tilde{\nu })}{∥r\left(\tilde{\nu }\right){∥}_{2}}. \end{array}$$

The resulting normalised spectra $\bar{r}\left(\tilde{\nu }\right)$ are shown in the right panel of Figure 5, the pure water spectra again in black and in blue the spectra of the sugary solutions. Clearly, the normalisation increases the measurements’ comparability, as it aligns the spectral baselines and thus creates a common zero reference. Moreover, the known vibrational fingerprint wavenumbers

(17) $$\begin{array}{cc} {\tilde{\nu }}_{\mathrm{gluc}} & =\{1034\;{\mathrm{cm}}^{-1},\;1078\;{\mathrm{cm}}^{-1}\}, \end{array}$$

(18) $$\begin{array}{cc} {\tilde{\nu }}_{\mathrm{fruc}} & =\{1063\;{\mathrm{cm}}^{-1},\;1080\;{\mathrm{cm}}^{-1}\} \end{array}$$

of glucose and fructose, highlighted in yellow, can easily be confirmed.

**Figure 6:**
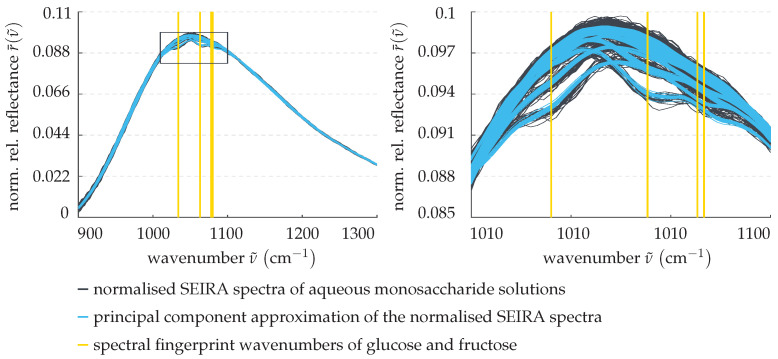
Pre-processing step 2: approximation of the SEIRA spectra by the four main principal components identified in the training data. (**Left panel**) normalised SEIRA spectra and their principal component approximation. (**Right panel**) magnification of the boxed area in the left panel.

Subsequently, the measurement data’s dimensionality is to be reduced and relevant patterns are to be isolated. To do so, a principal component analysis [25] of the normalised training data are performed. Mathematically speaking, this means centering the data and changing its basis by projecting it into the eigenspace of its covariance matrix. The SEIRA spectra are then given as linear combinations of the eigenvectors (principal components) weighted by their associated eigenvalues (scores). While principal components rarely have a physical interpretation, they each represent a pattern found in the data, for instance, they replicate the plasmonic resonance or exhibit local extrema at the spectral fingerprint wavenumbers of interest. Principal components associated with less dominant patterns, on the other hand, can be omitted from the superposition in order to decrease the data’s dimensionality and low-pass filter some of the measurement noise. Preliminary studies by Kühner et al. [23], as well as our own empirical investigations, have shown that, to model the SEIRA spectra, the four main principal components suffice. This is showcased in Figure 6, where the normalised example spectra of aqueous monosaccharide solutions previously introduced in Figure 5 are approximated by these.

Clearly, the resulting curves are much smoother and yet retain their general shape and features. This can be seen especially well on the right, where the spectral interval boxed in on the left is magnified. Evidently, the critical spectral dips at the fingerprint wavenumbers, and therefore the information about the monosaccharide levels, are preserved.

**Figure 7:**
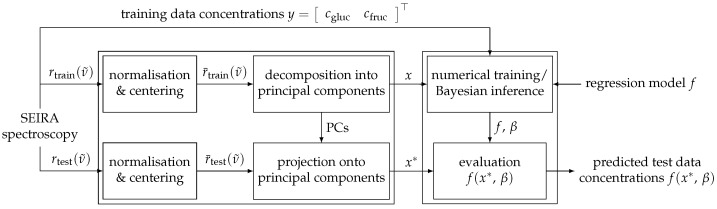
Graphical abstract of the signal processing routine performed in this paper: The measured data are split into training and test samples, normalised with respect to the $L^{2}$-norm and centered. Subsequently, the data are decomposed into or projected onto its principal components and passed on to the various regression models which finally output the predicted glucose and fructose concentrations in the sample.

The prominence of each principal component pattern in a single given sample is encoded by its respective score, which, in return, characterises a SEIRA spectrum in terms of that pattern. Accordingly, these scores are ideally suited as input data for the regression model. Therefore, in this paper, the scores $\left\{s_{i}\;|\;i=1\ldots 4\right\}$ of the first four principal components identified in the normalised training data are used as input vector

(19) $$\begin{array}{cc} x & ={\left[\begin{array}{ccc} x_{1} & \ldots & x_{4} \end{array}\right]}^{⊤} \end{array}$$

(20) $$\begin{array}{cc} \; & ={\left[\begin{array}{ccc} s_{1} & \ldots & s_{4} \end{array}\right]}^{⊤}\in R^{p=4} \end{array}$$

for the regression analysis. This implies that the test data, as well as any further measurement, have to be normalised, centered and projected onto these principal components. In summary, this yields the algorithm structure schematically outlined in Figure 7, which visualises the different processing of the training and test data as described in this section.

## 4. Results

In this section, the implementation of the various regression algorithms presented in Section 2 is addressed and their performance is demonstrated for the measurement series given in Section 3. Since machine learning methods lack an inherent physical interpretation of the data, to ensure the validity of the results, predicted negative concentrations are set to zero. Moreover, to robustify the regression against outliers and measurement noise, it is averaged over the predictions of five related measurements. Finally, the results are discussed and put into perspective.

**Remark** **2.** *Note that, in this section, all figures and plots share the same structure: To visualise our results, we employ error scatter plots. These show for each measurement the model prediction error e=y−f(x,β) in g/L as a function of the actual concentration y in g/L as a dark blue circular marker. More precisely, the top panel always shows the deviation in the glucose level estimate, while the lower panel shows accordingly the deviation of the estimate of the fructose level in the sample.*

### 4.1. Cascade-Forward Neural Network

For the regression analysis subject to this paper, we employ a CFNN with two hidden layers containing $h_{1}=40$ neurons in the first and $h_{2}=8$ neurons in the second one, respectively. Considering the weights and biases, this results in 666 tunable network parameters, i.e., $\beta \in R^{666}$. Furthermore, we use the popular sigmoid activation function

(21) $$\begin{array}{c} \phi \left(u\right)=\frac{1}{1+e^{-u}}. \end{array}$$

**Figure 8:**
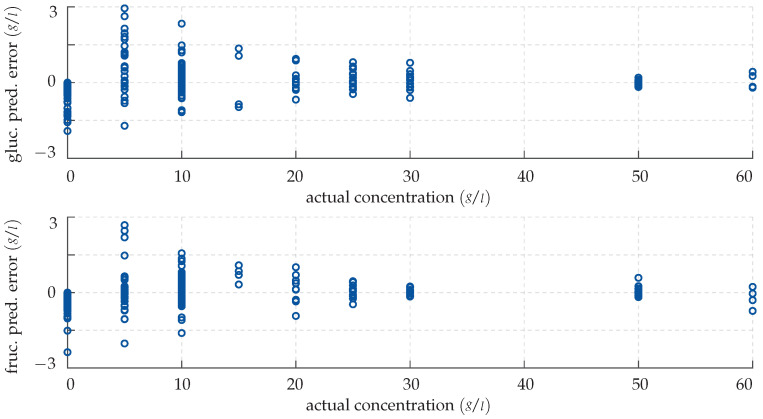
Prediction error made by the CFNN for the training data. (**Top panel**) deviation in the predicted glucose concentration plotted over the actual concentration. (**Bottom panel**) deviation in the predicted fructose concentration plotted over the actual concentration.

The network is trained with the computationally efficient, gradient-based Adam optimisation algorithm [49]. The goal of the training is to fit the network as closely as possible to the training data, i.e., to optimise the network’s predicted output for the given measurements. A fully trained model can therefore be benchmarked by re-evaluating it for some already known training samples. The results of this re-substitution are shown quantitatively with the error plot in Figure 8.

**Figure 9:**
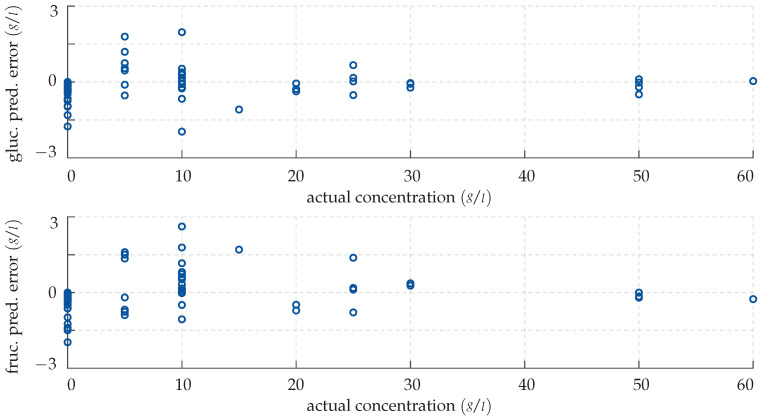
Prediction error made by the CFNN for the test data. (**Top panel**) deviation in the predicted glucose concentration plotted over the actual concentration. (**Bottom panel**) deviation in the predicted fructose concentration plotted over the actual concentration.

Clearly, the network’s output does not perfectly match the actual monosaccharide levels; however, in most cases, the predictions are quite accurate nonetheless. In fact, the mean absolute deviation of the network’s output for known input vectors is quite small with 0.22 g/L, which corresponds to 5.73%. This suggests that the training was successful and that the network has been adapted to the system, even though overfitting cannot be excluded at this point. The CFNN’s performance is successively demonstrated by inputting the previously unknown test data. Again, the results are visualised with an error plot, see Figure 9.

Qualitatively, the network replicates its good performance for the test data and provides very precise predictions, although some small deviations cannot be avoided. This leads to the conclusion that the network has not been over-fitted to the training data and is able to generalise. Note that the concentrations are over- and underestimated equally often; no general rule can be deduced from this. Furthermore, as can be seen, the network struggles with solutions of low monosaccharide concentrations: While the predictions are highly accurate from 25 g/L upwards, they are slightly less so below. This reduced sensitivity can be attributed to a decreased signal-to-noise ratio, i.e., the significant spectral fingerprint dips are not as pronounced and therefore more difficult to discriminate from measurement noise. A benchmark value taking this correlation into account is the mean relative deviation, which for the CFNN is 5.3% for the glucose and 7.82% for the fructose prediction, respectively. Note, however, that a respective maximum absolute error of 1.97 g/L in the glucose and 3.16 g/L in the fructose prediction is not surpassed.

### 4.2. Gaussian Process Regression

As mentioned in Section 2.2, in a Gaussian process regression, the kernel and mean function encode prior assumptions about the underlying system. Regarding the inverse problem of SEIRA sensing, we expect the functional dependence of the monosaccharide concentrations on the principal component decomposition of the normalised spectral measurement data to be roughly polynomial and smooth. This is in line with the findings of Schuler et al. [26]. Consequently, we employ the infinitely differentiable radial basis function kernel (sometimes referred to as squared exponential kernel)

(22) $$\begin{array}{c} k_{\mathrm{RBF}}\left(x,\;x^{\prime }\right)=\sigma^{2}\exp\left(-\frac{{\left(x-x^{\prime }\right)}^{2}}{2l^{2}}\right) \end{array}$$

and the second order mean function ansatz

(23) $$\begin{array}{c} \mu \left(x\right)=\left[\begin{array}{c} w_{0}^{\mathrm{gluc}}+\sum_{i=1}^{4}w_{i}^{\mathrm{gluc}}x_{i}+\sum_{i=1}^{4}\sum_{j=1}^{i}w_{ij}^{\mathrm{gluc}}x_{i}x_{j}\\ w_{0}^{\mathrm{fruc}}+\sum_{i=1}^{4}w_{i}^{\mathrm{fruc}}x_{i}+\sum_{i=1}^{4}\sum_{j=1}^{i}w_{ij}^{\mathrm{fruc}}x_{i}x_{j} \end{array}\right]. \end{array}$$

**Figure 10:**
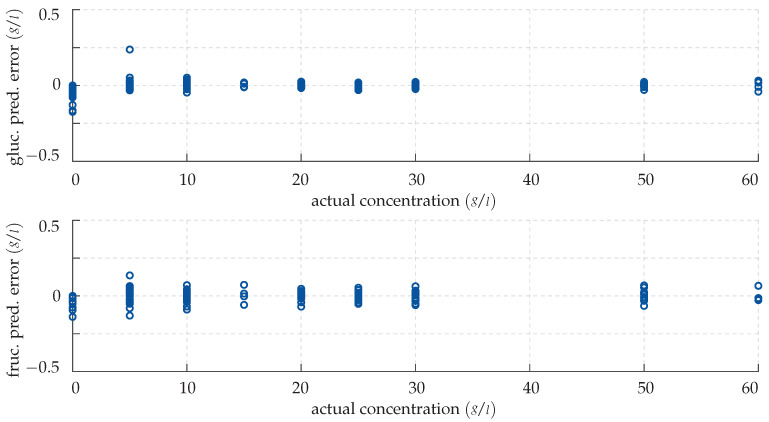
Prediction error made by the GPR for the training data. (**Top panel**) deviation in the predicted glucose concentration plotted over the actual concentration. (**Bottom panel**) deviation in the predicted fructose concentration plotted over the actual concentration.

Note that this does not necessarily imply that the Gaussian process regression model operates in a quadratic feature space. The hyper parameters $\sigma ,\;l\in R\;\;w^{\mathrm{gluc}},\;w^{\mathrm{fruc}}\in R^{15}$ as well as the measurement noise variance $\sigma_{n}\in R$ are optimised numerically in advance, before the predictive posterior distribution is inferred. Bayesian inference differs from neural network training in so far, that all available training data are explicitly exploited for each single prediction. This implies that a GPR yields highly accurate predictions for known inputs, as showcased in Figure 10.

Analogously to Figure 8, the discrepancies in the estimated glucose and fructose levels are plotted over the actual concentrations in the training samples. In contrast to the predictions obtained by the neural network in Section 4.1, however, the GPR matches the actual sugar levels almost perfectly with a mean absolute deviation of 0.009 g/L (0.22%).

**Figure 11:**
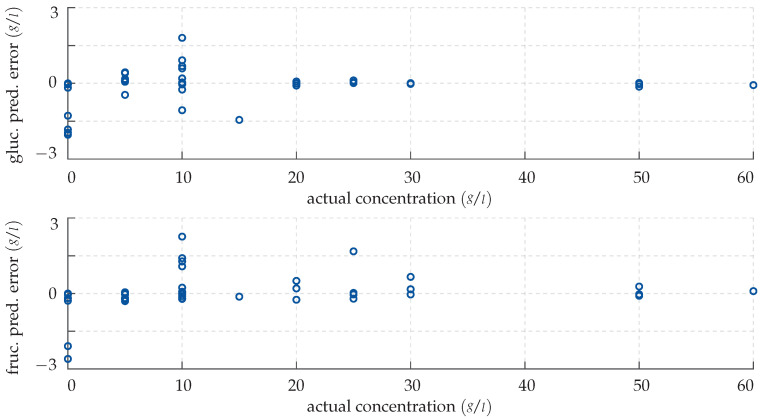
Prediction error made by the GPR for the test data. (**Top panel**) deviation in the predicted glucose concentration plotted over the actual concentration. (**Bottom panel**) deviation in the predicted fructose concentration plotted over the actual concentration.

As basis for a quantitative assessment of the performance of the GPR for the unknown test inputs consider the error plot in Figure 11. Clearly, like the CFNN, the GPR is more reliable and precise in detecting larger amounts of specimen. However, unlike the neural network, the predictions for lower monosaccharide concentrations are overall rather accurate as well. This is reflected in the GPR’s small mean relative deviations of 2.28% and 2.8% for the predicted glucose and fructose levels, respectively, which attest to the method’s high sensitivity.

## 5. Discussion and Outlook

Overall, the presented machine learning methods of regression generalise well from the training data and provide both accurate and reliable estimates of the glucose and fructose concentrations in the analysed samples. However, it stands out that both approaches perform slightly but clearly better at predicting glucose concentrations. This might be attributed to the design of the gold nanoantennas in the SEIRA sensor flow cell: Since their geometry was chosen to match the plasmonic resonance to the spectral fingerprint of glucose, measurements of the fructose level suffer a lower signal-to-noise ratio, which, in return, compromises the accuracy of the concentration estimates.

In the following, consider the key benchmark values reached by the different algorithms collected in Table 3. To allow for a more well-rounded comparison and a thorough evaluation, the scores achieved by classic methods of regression (i.e., linear/polynomial regression, support vector regression) and by the optimisation-based method by Schuler et al. [26] are listed as well.

In total, three key benchmark values are computed, each evaluating a different aspect of the prediction accuracy. Respectively, the best values are printed in bold. The maximum absolute deviation allows for an evaluation of the algorithms’ worst-case performance and therefore quantifies the regression models’ ability to generalise from the training data. The mean deviation, in contrast, provides a measure for the reliability and consistency of the predictions. Here, for the sake of completeness, both the absolute and the relative mean error are given. Finally, as an overall score, the root mean squared (RMS) error combines the distinct aspects of the other benchmarks: On the one hand, the error over the entire measurement series is taken into account; on the other hand, larger deviations are penalised more heavily.

Linear regression represents a first, naive approach in many cases. For the inverse problem of SEIRA glucose sensing, however, it clearly does not provide an adequate solution. In fact, with a mean relative deviation of roughly 40% and errors reaching up to 21.45 g/L, many predictions are effectively mere educated guesses and not sufficiently reliable. This underlines that the correlation between the monosaccharide concentrations and the measured spectrum is nonlinear. Accordingly, the second order polynomial regression achieves significantly better results, as projecting the input data into a quadratic feature space partially accounts for this nonlinearity. Note that, with an RMS error of 1.73 g/L compared to the polynomial regression’s 1.81 g/L, the support vector regression performs similarly well. Considering the related mathematical formulations of both regression strategies, it is reasonable to assume that the latter operates in a near-quadratic feature space, too. A quadratic relationships is also assumed in the selection of the Ansatz functions in the optimisation-based method by Schuler et al. [26]. Unsurprisingly, this approach surpasses the other two by only a small margin.

Overall, the Gaussian process regression model tops the ranking ahead of the cascaded neural network. Both approaches clearly outperform all classic methods of regression, proving highly reliable and producing basically no outliers. This implies, that both successfully compensate the nonlinearity in the data. However, with its relative and absolute mean deviation less than half that of the runner-up, the GPR stands out in particular for its consistency and continuous accuracy. As mentioned in Section 4, especially at predicting lower monosaccharide concentrations, the GPR performs better than the neural network. A possible explanation for this is that the probabilistic setup inherently takes uncertainties in the system like measurement noise explicitly into account and is therefore able to better filter the information. Considering that most sensors employ linear calibration models, the aspects above imply that Gaussian process regression has a high potential not only as a solution to the inverse problem of SEIRA glucose sensing, but for sensor calibration in general. To investigate this hypothesis and explore the transferability of our findings, we suggest a calibration of other biosensing systems by GPR. Future studies could also evaluate the use of Bayesian neural networks: As an analytic solution, the Bayesian conditioning of the predictive posterior distribution in a GPR makes sure that all available training data are explicitly used. While this in itself is an advantage, it comes at the expense of computability. This constitutes a key difference compared to neural networks: It is to be expected that training-based approaches would benefit the most from a larger batch of training data, as, on the one hand, numerical effects would have less impact and, on the other hand, the feasibility of a globally optimal adaptation would increase. Bayesian neural networks theoretically exploit the advantages of both regression strategies and might therefore offer further improvements.

Clearly, in its current configuration, the SEIRA glucose sensor by Kühner et al. [23] is in an early stage of development and is not competing with state of the art glucose monitoring devices approved for medical use. However, by pre-processing the measurement data as described in Section 3.1 and employing modern machine learning algorithms for signal analysis, a roughly 60% lower RMS error than Schuler et al. [26] and thus significant improvements on previous methods could be achieved. For the further development of the sensor, we suggest to focus more heavily on the critical concentrations of below 5 g/L and to explore different nanoantenna geometries.

## Figures and Tables

**Table 1:** Single monosaccharide samples: Number of analysed samples for each concentration of either glucose or fructose in an aqueous solution.

Monosaccharide Concentration in the Sample in g/L	Number of Samples
5	4
10	8
20	2
25	2
30	2
50	3

**Table 2:** Double monosaccharide samples: Number of analysed samples for each composition of concentrations of glucose and fructose in an aqueous solution.

Concentration of Glucose in the Sample in g/L	Concentration of Fructose in the Sample in g/L	Number of Samples
5	5	2
5	10	1
10	5	1
10	10	3
10	15	1
15	10	1
10	20	2
20	10	2
25	25	1
30	60	1
60	30	1
50	50	1

**Table 3:** Rating scores for various monosaccharide concentration estimation methods.

Method of Estimation	Maximum Absolute Deviation g/L	Mean Deviation Abs. g/L, Rel. %	RMS Error g/L
Cascade-forward neural network	3.16	0.29	0.57
		6.56	
Gaussian process regression	2.6	0.15	0.47
		2.54	
Linear regression	21.45	4.05	5.93
		39.94	
2nd order polynomial regression	8.37	1.11	1.81
		17.48	
Support vector regression	8.11	0.61	1.73
		14.78	
Schuler et al. [26]	4.29	0.71	1.23
		5.15	

## Data Availability

Not applicable.

## Appendix A. Technical Specifications and Software

For the processing of the recorded data, computations, and visualisations both Python and MATLAB${}^{®}$ were used.

**Table A1:** Technical equipment used for recording the measurements.

gold nanoantennas	length =3500 nm, width, thickness =100 nm,
	2 nm chromium adhesion layer underneath,
	periodicity in *x*-direction =4500 nm,
	periodicity in *y*-direction =3000 nm,
	fabricated via electron beam lithography (EBL)
FTIR spectrometer	Bruker VERTEX 80
	Bruker Optik GmbH, 76275 Ettlingen, Germany
Optical microscope	Bruker Hyperion 2000, Schwarzschild objective
	15-fold magnification, NA=0.4
	Bruker Optik GmbH, 76275 Ettlingen, Germany
detection	nitrogen-cooled mercury cadmium telluride (MCT)
	detector, measurement spot 90 μm×90 μm
